# A Machine Learning Approach to Achieving Energy Efficiency in Relay-Assisted LTE-A Downlink System †

**DOI:** 10.3390/s19163461

**Published:** 2019-08-08

**Authors:** Hammad Hassan, Irfan Ahmed, Rizwan Ahmad, Hedi Khammari, Ghulam Bhatti, Waqas Ahmed, Muhammad Mahtab Alam

**Affiliations:** 1 School of Electrical Engineering and Computer Science, National University of Sciences & Technology (NUST), Islamabad 44000, Pakistan; 2 Department of Electrical Engineering, Higher Colleges of Technology, Ruwais Campus 12389, UAE; 3 College of Computers and Information Technology, Taif University, Taif 21974, Saudi Arabia; 4 Pakistan Institute of Engineering and Applied Sciences (PIEAS), Islamabad 45650, Pakistan; 5 Thomas Johann Seebeck Department of Electronics, Tallinn University of Technology, Tallinn 19086, Estonia

**Keywords:** machine learning, LTE-A, energy efficiency, resource block allocation, bisection based optimal power allocation, water filling algorithm, proportional rate constraint

## Abstract

In recent years, Energy Efficiency (EE) has become a critical design metric for cellular systems. In order to achieve EE, a fine balance between throughput and fairness must also be ensured. To this end, in this paper we have presented various resource block (RB) allocation schemes in relay-assisted Long Term Evolution-Advanced (LTE-A) networks. Driven by equal power and Bisection-based Power Allocation (BOPA) algorithm, the Maximum Throughput (MT) and an alternating MT and proportional fairness (PF)-based SAMM (abbreviated with Authors’ names) RB allocation scheme is presented for a single relay. In the case of multiple relays, the dependency of RB and power allocation on relay deployment and users’ association is first addressed through a *k*-mean clustering approach. Secondly, to reduce the computational cost of RB and power allocation, a two-step neural network (NN) process (SAMM NN) is presented that uses SAMM-based unsupervised learning for RB allocation and BOPA-based supervised learning for power allocation. The results for all the schemes are compared in terms of EE and user throughput. For a single relay, SAMM BOPA offers the best EE, whereas SAMM equal power provides the best fairness. In the case of multiple relays, the results indicate SAMM NN achieves better EE compared to SAMM equal power and BOPA, and it also achieves better throughput fairness compared to MT equal power and MT BOPA.

## 1. Introduction

Green Radio communication has received a lot of attention in the past few years with an aim to decrease the carbon foot print of wireless networks. It has been estimated that nearly 70% of the energy being used by cellular operators is on the radio part [1] and around 9% of the global CO${}_{2}$ emission is from the communication systems [2]. In addition, one of the main concerns is the User Equipment (UE) battery, which has not shown progression at par with the Radio Access Technology (RAT). This phenomena is highly visible for the cell edge users that despite spending higher energy (due to high pathloss shadow fading and adjacent cell interference) are unable to achieve fair share of the radio resources. In this context, Green communications employing cooperative and fair resource allocation techniques can help in reducing the carbon footprint and increasing Energy Efficiency (EE).

Most of the existing wireless systems use Orthogonal Frequency Division Multiple Access (OFDMA) to distribute radio resources among UEs. One of the existing RAT to use OFDMA is Long Term Evolution-Advanced (LTE-A), which has a similar structure to its predecessor LTE. In LTE, each Resource Block (RB) is a time frequency grid element. The basic RB structure contains 15 subcarriers of 12 KHz each and a 10 ms frame. Each frame is subdivided into 10 subframes of 1 ms and each subframe is further divided into 2 slots of 0.5 ms each. Each slot may contain 6 or 7 OFDM symbols depending on a normal or an extended cyclic prefix. The RB allocation can be changed after every Transmission Time Interval (TTI) based upon channel conditions or RB allocation algorithm. OFDMA offers flexibility of RBs Allocation to tailor user and network requirement, such as throughput, fairness, Energy EfficiencyEE, and Spectral Efficiency (SE). For example, in order to support higher peak data rates Carrier Aggregation (CA) is introduced to obtain wider bandwidth. Compared to LTE, CA in LTE-A can support maximum 5 adjacent/non-adjacent component carriers of maximum 20 MHz to achieve 100 MHz bandwidth.

In addition, LTE-A allows Layer 3 (L3) relays to be incorporated in the network that can decode and forward the data to a UE [3]. This cooperative communication addresses EE and throughput of the cell edge users by providing channel diversity. As the network can only accommodate finite relays, their placement is, therefore, crucial to manage the overall throughput. The RB allocation between direct link that is Base Station (BS)–UE and two hop link that is (BS)–Relay Node (RN)–UE can be done independently or in a shared manner. In [4], the authors have presented a thorough comparison of basic RB allocation schemes, which are Round Robin (RR), Proportional Fairness (PF), Maximum Throughput (MT), and Maximum Minimum (MM); they presented an alternating MT and PF based resource allocation scheme SAMM (abbreviated with Authors’ names) without considering any relay. The paper considered LTE system with a basic RB structure [5], 5 MHz bandwidth with fixed 10 users that are uniformly placed from the BS. These schemes have been compared in terms of sum throughput, individual user throughput and fairness based on JFI (Jains fairness index). The SAMM scheme provides a better tradeoff between throughput and fairness. Cell edge users show some throughput gains due to proportional fairness, however, this scheme fails to address EE. Authors in [6] considered EE for generic OFDMA based downlink system. They presented Bisection based Optimal Power Allocation (BOPA) algorithm for a given RB assignment. The BOPA works as an iterative approach based on water filling principle. This work uses equal power allocation among users for initial users’ rate calculation, whereas, a modified algorithm in [7] uses equal power per resource block.

In [8], the authors proposed a quality of service (QoS) aware optimization problem for relay-based multi-user cooperative OFDMA uplink system. The main goal is to find optimal solutions for relay selection, power allocation and subcarrier assignment that maximize the system throughput. Aiming to support and attain the green wireless LTE network, an energy-efficient resource allocation scheduler with QoS aware support for LTE network is proposed in [9]. The authors of [10] proposed a two-stage method to solve the inter-cell interference problem. In the first stage, the subcarrier allocation and time scheduling are jointly conducted with sequential users’ selection and without considering the interference. The power control optimization is left to the second stage, using a geometric programming method.

In [11], energy efficient resource block and power allocation optimal and low complexity suboptimal schemes are presented for OFDMA relay-assisted downlink. Authors use fractional programming to make the non-linear mixed integer problem to convex subtractive problem. In order to reduce the computational complexity of the optimal solution, they present two-stage RB allocation and transmission power control algorithms. The system model of this paper is similar to our model but they use relays (small eNB in that paper) with frequency reuse factor of one, and the users employ maximal ratio combining to maximize the received signal-to-noise ratio (SNR). In our case, we use multiplexing gain instead of the diversity gain at users’ end by exploiting the knowledge of their location in relay selection and users association algorithm. We have compared our proposed schemes with the low complexity energy-efficient resource block and power allocation (LERPA) algorithm 3 and 4 of [11].

Artificial intelligence techniques can be used in highly dynamic and stringent constraint Next-Generation networks. Since machine learning is a most promising technique of artificial intelligence, it can be directly/indirectly employed to achieve the goals of 5 G in cognitive radios, massive multiple-input multiple-output (MIMO), hybrid beamforming, femto/small cells, smart grid, wireless power transfer, device-to-device communications, non-orthogonal multiple access (NOMA) etc. [12]. This paper [12] gives an overview of the applications of machine learning in Next-Generation wireless networks. Specifically, supervised learning techniques are suitable for massive MIMO channel estimations and spectrum sensing, unsupervised learning could be helpful in users grouping and clustering; and reinforcement learning can be applied in resource allocation problems.

A detailed review on existing techniques and methods have been provided in [13]. For example, in [14], a cooperative Q-learning approach was applied as an efficient approach to solve the resource allocation problem in a multi-agent network. The quality of service QoS for each user and fairness in the network are taken into account and more than a four-fold increase in the number of supported small cells. The authors in [15], proposed a machine learning framework for resource allocation to determine the optimal or near-optimal solutions based on the learning of the most similar historical scenario.

In paper [16], the authors proposed an approximated solution to a wireless network capacity problem using flow allocation, link scheduling, and power control. The Support Vector Machine (SVM) was used to classify each link to be assigned maximal transmit power or be turned off, whereas, the deep belief networks (DBNs) computes an approximation of the optimal power allocation. Both learning approaches have been trained on offline computed optimal solutions. A novel resource allocation method using deep learning to squeeze the benefits of resource utilization was developed in [17]. It was reported that when the channel environment is changing fast, the deep learning method outperforms traditional resource optimization methods. The resource allocation is to be optimized by a convolutional neural network using channel information. A similar problem has been explored in [18] that use Upper Confidence Bound learning for Greedy Maximal Matching (GMM) when the channel statistics are unknown. Since the subchannel and power allocation problem is a non-convex combinatorial problem, the optimal solution of the subchannel and power allocation problem requires an exhaustive search over all possible combinations of subchannels and power levels. In order to train the deep neural network (DNN) for an optimal solution, Ref. [19] utilizes the genetic algorithm to get the training data for DNN. It shows that the prediction accuracy increases with the size of dataset and the number of hidden layers. A four-step reinforcement learning based intercell interference coordination (ICIC) scheme is presented in [20]. The users selection, resource allocation, power allocation, and retransmit packet identification are handled by reinforcement learning to reduce the intercell interference.

However, to the best of our knowledge no available literature discusses LTE-A with L3 relays for SE and EE consideration. In this work,

- We present an energy efficient algorithm based on SAMM and BOPA for LTE-A system with a L3 relay. Performance evaluation in terms of throughput, fairness, power consumption, SE and EE is shown between two best performing schemes i.e., MT and SAMM considering equal power and BOPA.
- Considering the practical deployment, where there may be more than one relay supporting the cell edge users, we devise a clustering strategy to obtain near optimal placement of L3 relays and users’ association.
- In a multiple relay scenario, to optimize EE and reduce computational complexity of running algorithm every TTI, we present a two step machine learning process that uses both the SAMM and BOPA approach for resource and power allocation of the cell users. The proposed approach is compared to MT equal power, MT BOPA and SAMM equal power in terms of users’ throughput and EE.

A complete list of notations used in this paper is given in Table 1.

Rest of the paper is organized as follows: system model is described in Section 2, algorithms and performance for MT, SAMM and BOPA with single relay network are given in Section 3. Multiple relay users’ association and deployment with machine learning based power and RB allocation for SAMM is presented in Section 4. Complexity analysis is given in Section 5, followed by the conclusions in Section 6.

## 2. System Model

**Figure 1:**
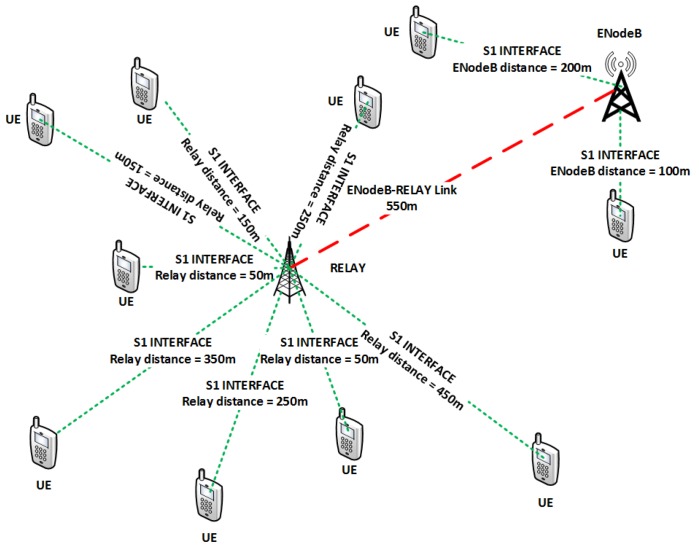
Topology.

We consider a two-tier LTE-A system with a BS supported by L3 relays as shown in Figure 1. The relays are assumed to be In-band type 1b [3] and full duplex, placed in the center of BS to the most distant user. A total of *K* users and *N* RBs are considered with users placed at a uniform distance from BS. The total powers of BS and RN are denoted by $P_{total}^{BS}$ and $P_{total}^{RN}$, respectively. The LTE-A system uses OFDMA transmission in the downlink. Let the system bandwidth is *B* with *N* number of RB, then, $W=\frac{B}{N}$ is the bandwidth of one RB. We express the channel gains $g_{k,n}^{Direct\_link}$ and $g_{k,n}^{Relay\_link}$ for user *k* where k∈K={1…,K} on RB *n* where n∈N={1…,N} for BS and RN respectively. Practically, the channel gain depends upon various factors, including thermal noise at receiver, receiver noise figure, antenna gains, distance between transmitter and receiver, path loss exponent, log normal shadowing and fading. Therefore, for all the links, we can write

(1) $$g_{k,n}=-\varrho -\phi 10\log_{10}d_{k}-\zeta_{k,n}+10\log_{10}h_{k,n}$$

In the above equation, ϱ (83.46 dB) is a constant depending upon thermal noise at receiver, receiver noise figure, and antenna gains, ϕ is path loss exponent, $d_{k}$ is the distance in Km from UE *k* to the BS/relay, $\zeta_{k,n}$ (10.5 dB) is shadowing parameter modeled by a normally distributed random variable with standard deviation 8 dB, and $h_{k,n}$ corresponds to the Rayleigh fading channel coefficient of user *k* in subchannel *n* [21].

The throughput of user *k* is given by,

(2) $$R_{k}=\left\{\begin{array}{cc} \frac{1}{2}\frac{B}{N}\sum_{n=1}^{N}\mu_{k,n}\log_{2}\left(1+SNR_{k,n}\right), & \mathrm{Access}\;\mathrm{link}\;\mathrm{users}\\ \frac{B}{N}\sum_{n=1}^{N}\mu_{k,n}\log_{2}\left(1+SNR_{k,n}\right), & \mathrm{Direct}\;\mathrm{link}\;\mathrm{users} \end{array}\right.$$

where the factor 1/2 in access link shows the two time-slots transmission from BS-RN and RN-UE, and $\mu_{k,n}$ is the binary variable such that $\mu_{k,n}=1$ when RB *n* is allocated to the user *k*, $SNR_{k,n}$ is the maximum average signal-to-noise ratio for user *k* between direct and relay links. Let $SNR_{k,n}^{Direct\_link}$ be the signal-to-noise ratio for user *k* via Direct Link, and $SNR_{k,n}^{Relay\_link}$ be the signal-to-noise ratio for user *k* via Relay Link, then, the $SNR_{k,n}$ is given as

(3) SNRk,n=max(E{SNRk,nDirect_link},E{SNRk,nRelay_link}),

(4) $$SNR_{k,n}^{Direct\_link}=\frac{p_{k,n}^{BS}g_{k,n}^{Direct\_link}}{N_{0}\frac{B}{N}},$$

(5) SNRk,nRelay_link=min(SNRk,nbackhaul_link,SNRk,naccess_link),

where, $SNR_{k,n}^{backhaul\_link/access\_link}$ is

(6) $$SNR_{k,n}^{backhaul\_link/access\_link}=\frac{p_{k,n}^{BS/RN}g_{k,n}^{backhaul\_link/access\_link}}{N_{0}\frac{B}{N}}$$

The Energy Efficiency EE in terms of bits/s/Watts can be expressed as

(7) $$EE=\frac{\sum_{k=1}^{K}\sum_{n=1}^{N}\mu_{k,n}\log_{2}\left(1+SNR_{k,n}\right)}{\sum_{k=1}^{K}\sum_{n=1}^{N}p_{k,n}}.$$

The EE optimization problem for the above scenario can be written as

(8) $$\begin{array}{cc} \mathrm{maximize} & EE\\ \mathrm{subject}\;\mathrm{to} & \sum_{k=1}^{K}\sum_{n=1}^{N}p_{k,n}\leq P_{total}\\ & p_{k,n}\geq 0,\forall \;k,n\\ & \mu_{k,n}=\left\{0,1\right\},\forall \;k,n\\ & \sum_{k=1}^{K}\mu_{k,n}=1,\forall \;n\\ & R_{1}:R_{2}:\ldots \ldots \ldots R_{K}=\alpha_{1}:\alpha_{2}:\ldots \ldots \ldots \alpha_{K} \end{array}$$

where $\alpha_{k}$ is the proportional rate constraint [22]. We assume that channel state information (CSI) of all the users is known to the BS. Also, it is assumed that the RB allocation decision and assignment is done in less than channel coherence time so that CSI information can be used. This further puts constraints on the RB allocation algorithm complexity. The two-hop transmission to the RN users will be carried out in two TTI’s. In the first TTI, the BS will only send data to the RN users that are in close proximity of RN or have better RN-UE channel conditions than the direct link BS-UE. In the second TTI RN-UE data will be sent. BS will choose the path to the user (direct or via RN) with best channel coefficient in each TTI. The centralized scheduling minimizes the possibility of interference for In-band type of RNs. Frequency division duplexing ensures that the RN may handle backhaul data simultaneously with the access link data so that from the second TTI onwards backhaul BS-RN transmission is carried out simultaneously with the access link RN-UE transmission.

The LTE-A downlink is an OFDM based system which supports M-ary quadrature amplitude modulation (MQAM). We can use Equation (2) to calculate the throughput of user *k* on RB *n* for both direct and relay-link paths. The two paths provide channel diversity to increase the users and system level throughput. We use MT and SAMM criteria for RB allocation with equal power allocation to all RBs or BOPA as explained below.

## 3. Fairness-Aware Power and Resource Block Allocation with Single Relay LTE-A Network

There are several well-known resource allocation schemes for cellular systems, namely, round robin RR, maximize throughput MT, maximize the minimum throughput (max-min), and proportional fairness PF. An improved hybrid MT and PF scheme, SAMM is presented in [4]. We briefly summarize MT, PF, and SAMM, and then present our fairness-aware power and resource allocation algorithm.

### 3.1. Maximum Throughput

In a Maximize Throughput MT scheme, the aim is to maximize the sum throughput of the network. It assigns more RBs to the user which has better channel conditions on direct link or two hop link thereby adding more throughput to the system but its drawback is that users with the worst channel conditions are essentially ignored. The maximum throughput criterion in mathematical form is given as,

(9) $$D_{k}=\arg\max_{k}\left(R_{k}\right)$$

where $D_{k}$ is RB allocation matrix and $R_{k}$ is rate matrix.

### 3.2. Proportional Fairness

The proportional fairness based resource allocation schemes are widely used in practical wireless communication systems. In this scheme, the system allocates the resource to a user who has the maximum PF metric. The PF criterion in mathematical form is given as,

(10) $$D_{k}=\arg\max_{k}\;\frac{R_{k}\left(t\right)}{{\bar{R}}_{k}\left(t\right)}$$

where $R_{k}\left(t\right)$ is the throughput of user *k* at scheduling time *t*, and ${\bar{R}}_{k}\left(t\right)$ is the average user throughput (moving average) over a past window of length $T_{w}=1/\alpha$ [23], as

(11) $${\bar{R}}_{k}\left(t\right)=\alpha R_{k}\left(t-1\right)+\left(1-\alpha \right){\bar{R}}_{k}\left(t-1\right),$$

### 3.3. SAMM

In SAMM [4] PF and MT are run one after the other, i.e., in first TTI PF run for *K* users and in second TTI MT run for K−1 users ignoring the user with highest throughput in previous TTI. This results in maximizing fairness and throughput alternatively in each TTI.

### 3.4. BOPA Algorithm

Bisection based optimal power allocation BOPA Algorithm 1 allocates the power to the RBs assigned to a particular user. Given the RB allocation from MT or SAMM and throughput of each user at equal power allocated to all RBs we can calculate λ “rate parameter” as given below:

(12) $$\lambda_{k}=\frac{R_{1}}{\alpha_{1}}=\frac{R_{2}}{\alpha_{2}}=\ldots =\frac{R_{K}}{\alpha_{K}}$$

where $R_{k}$ is the rate of each user and $\alpha_{k}$ is proportional rate constraint set for fairness [6]. Optimal power allocation is water filling operation and obtained for single user as

(13) $${\hat{p}}_{k,n}=\max\left\{\frac{1}{\theta_{L}\ln2}-\frac{1}{g_{k,n}},0\right\}$$

where $\theta_{L}$ is Lagrangian multiplier and its value is chosen such that $R_{k}$ is satisfied. Hence, the user power can be expressed as $P_{k}\left(\lambda \alpha_{k}|D_{k}\right)$ and the total transmit power $P_{T}\left(\lambda \right)$ can be rewritten as

(14) $$P_{T}\left(\lambda \right)=\sum_{k\in K}P_{k}\left(\lambda \alpha_{k}|D_{k}\right)$$

EE can be given as user rate divided by power consumed to achieve that rate.

(15) $$EE\left(\lambda \right)=\frac{\lambda \sum_{k\in K}\alpha_{k}}{P_{T}},$$

and total transmit power is also limited by

(16) $$\lambda \leq \lambda_{\max}.$$

According to [24] if transmit power $P_{T}\left(\lambda \right)$ is strictly convex in rate then EE(λ) is quasi-concave, global optimal solution proof is given in the appendix of paper [22]

(17) $$f\left(\lambda \right)=P_{T}\left(\lambda \right)-\lambda \ln2\sum_{k\in K}\min_{n\in D_{k}}\left\{\frac{1+{\hat{p}}_{k,n}g_{k,n}}{g_{k,n}}\right\}\alpha_{k}$$

Bisection method is a simple and robust. Since the method brackets the root, it is guaranteed to converge. We apply BOPA on the RB allocation scheme SAMM, an alternating MT and PF scheme for the relay-assisted LTE-A for the optimal power allocation with the objective of maximizing the EE. In addition, we trained neural network with the dataset generated by the BOPA. Since power is a monotonically increasing function of the rate parameter λ, we apply bisection method on the following equation to find the root,

(18) $$P\left(\lambda \right)=\sum_{k}\sum_{n}\frac{2^{\lambda \alpha_{k}N}-1}{g_{k,n}}-P_{total}=0$$

**Algorithm 1** BOPA Algorithm
1:**Require**:${\hat{p}}_{k,n}$ is the optimal power allocation matrix.2:**Ensure**: Prior RB allocation through any algorithm and given as $D_{k}$.3:Getting all the λ then calculate $\lambda_{max}$ which gives the max energy Efficiency by substitution in Equation (6).4:Using $\lambda_{max}$ set user rate as $\alpha_{k}$$\lambda_{max}$, do water filling using Equation (13) and calculate f($\lambda_{max}$ ) based on Equation (17).5:**If** f ($\lambda_{max}$ ) ≥ 06:
**Return;** ${\hat{p}}_{k,n}$
7:**Else** Go to Step 9;8:
**End if**
9:Set $\lambda_{high}$ = $\lambda_{max}$, $\lambda_{low}$ = 0, $\lambda_{current}$ = $\lambda_{max}$ /210:**Repeat:** Set user rate according to $\alpha_{k}$$\lambda_{max}$, do water filling using Equation (13) and calculate f($\lambda_{max}$) based on Equation (17).11:**If** f ($\lambda_{current}$ ) > 012:Set $\lambda_{low}$ = $\lambda_{current}$13:**Else** Set $\lambda_{high}$ = $\lambda_{current}$14:
**End if**
15:Set $\lambda_{current}$ = $\lambda_{high}$ + $\lambda_{low}$ / 216:
**Return** ${\hat{p}}_{k,n}$
17:
**End if**



### 3.5. Performance Evaluation

A single cell is considered for generating simulations results. The cell consists of a BS, RN and UEs equipped with Omni-directional antennas. The throughput, energy and spectral efficiency is averaged over 1000 TTIs, with the duration of a TTI being 0.5 ms. The channel involves Raleigh fading and distance based path loss as shown in Figure 1. BS is located in the center of the cell coverage and most distant user is 1 Km distant from BS with RN in between at 0.55 Km. RN are In-band full duplex relays and bit error rate (BER) considered for MQAM modulation is $10^{-3}$. Table 2 below summarizes all simulation parameters used to derive results shown next.

**Figure 2:**
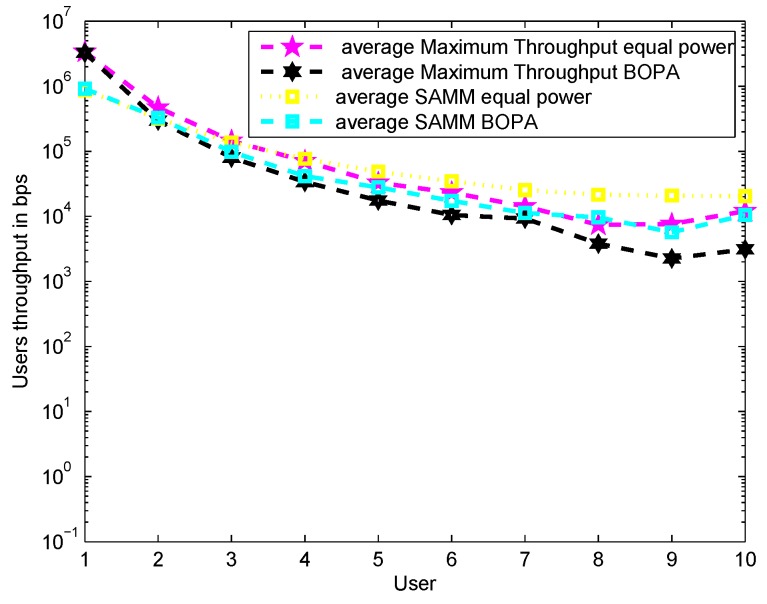
User vs. Throughput averaged over thousand times for SAMM and MT with equal power and BOPA.

**Figure 3:**
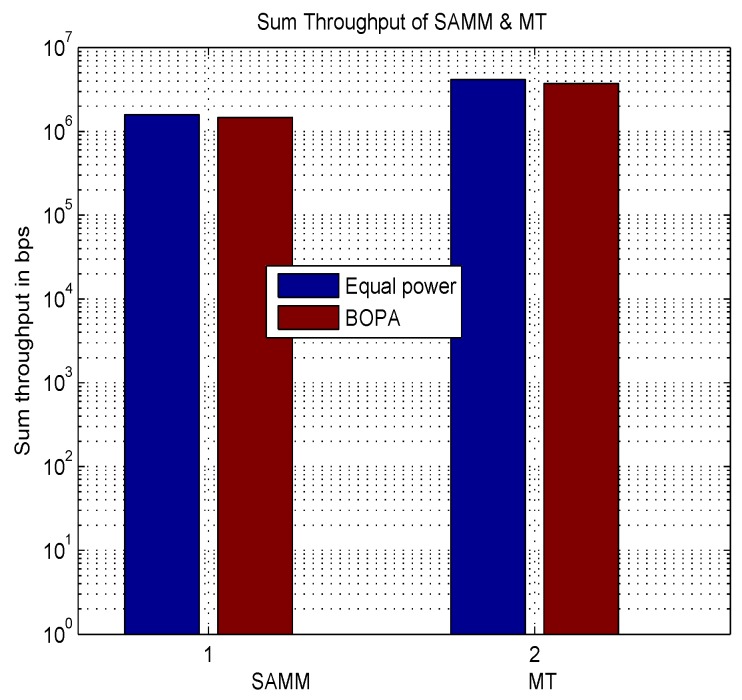
Sum throughput of all users.

Figure 2 shows the result of average throughput for MT and SAMM with equal power and BOPA based power allocation. It can be seen that SAMM curves remain on top of MT curves for most of the users due to inherent fairness which ensures all users get due share of RBs. However as evident from Figure 3 Sum throughput of MT is higher as compared to SAMM for overall averaged throughput of sum users due to channel exploitation of users with good channel conditions. This makes MT better than SAMM as BOPA has proportional rate constraint set for assigning user priorities.

**Figure 4:**
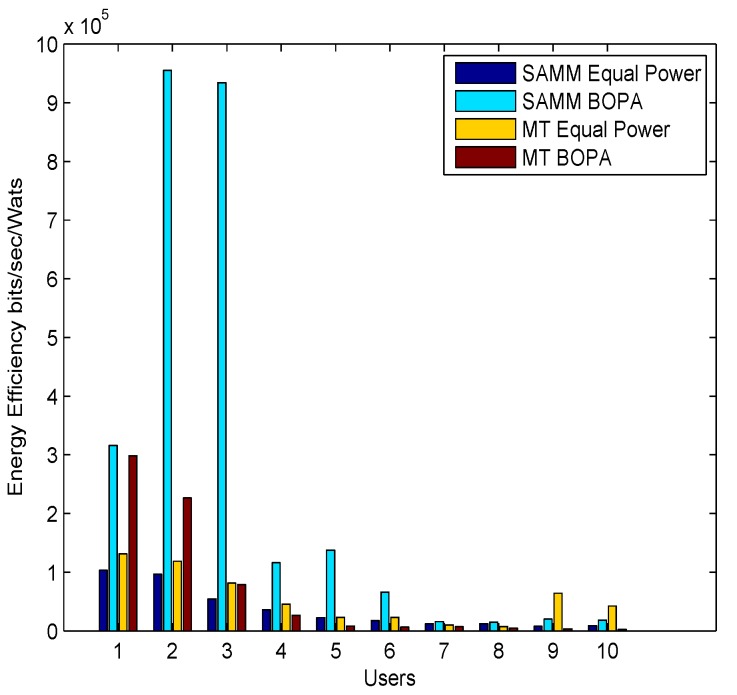
Users vs. Energy Efficiency (bits per second per watts) for all four schemes.

Figure 4 shows energy efficiency per user in bits per seconds per watts. SAMM BOPA outperforms for initial users and remains considerably lower for rest of the users. Whereas MT BOPA compared to all other schemes performs better for every user of the system with consistency due to convergence of BOPA to maximize throughput and minimize energy.

**Figure 5:**
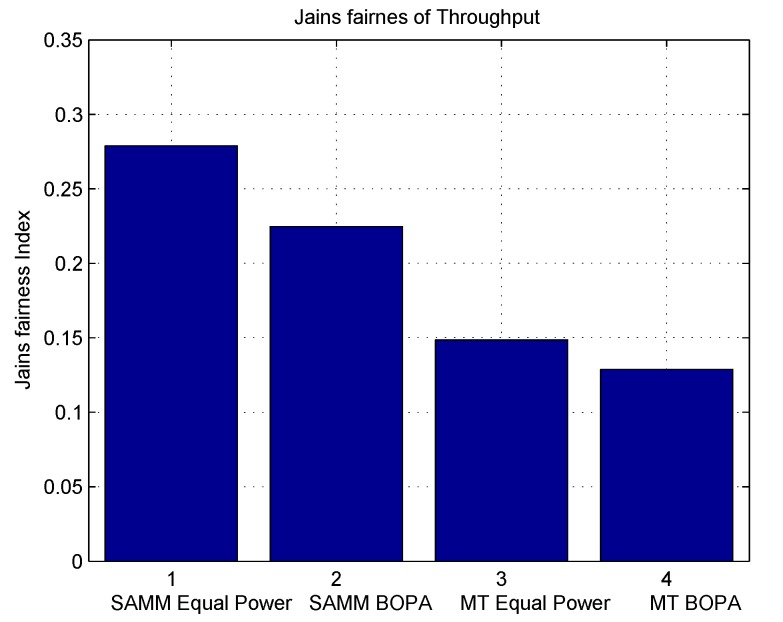
Jains fairness Index for Throughput.

**Figure 6:**
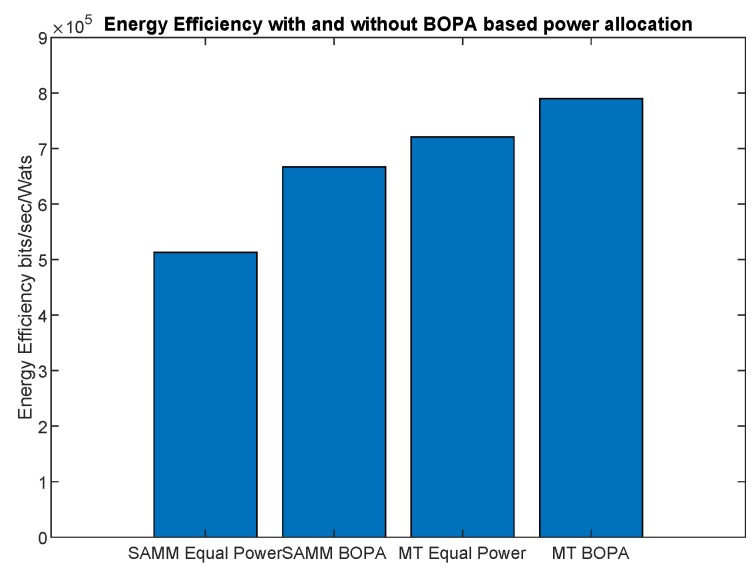
The system energy efficiency with and without BOPA based power allocation.

Figure 5 shows fairness Index using Jains fairness Index [25] using below equation

(19) $$FI=\frac{{\left(\sum_{k=1}^{K}r_{k}\right)}^{2}}{K\sum_{k=1}^{K}{r^{2}}_{k}},$$

where $r_{k}$ can be throughput or EE. Figure 5 shows SAMM has better fairness in terms of throughput due to PF in its algorithm. Figure 6 depicts the system’s energy efficiency EE with and without power allocation. The BOPA-based power allocation algorithm allocates the available power to the RB to maximize the energy efficiency EE, therefore, both MT-BOPA and SAMM-BOPA outperforms their corresponding MT and SAMM schemes with equal power allocation.

## 4. Fairness-Aware Machine Learning Based Power and RB Allocation with Multiple Relays

**Figure 7:**
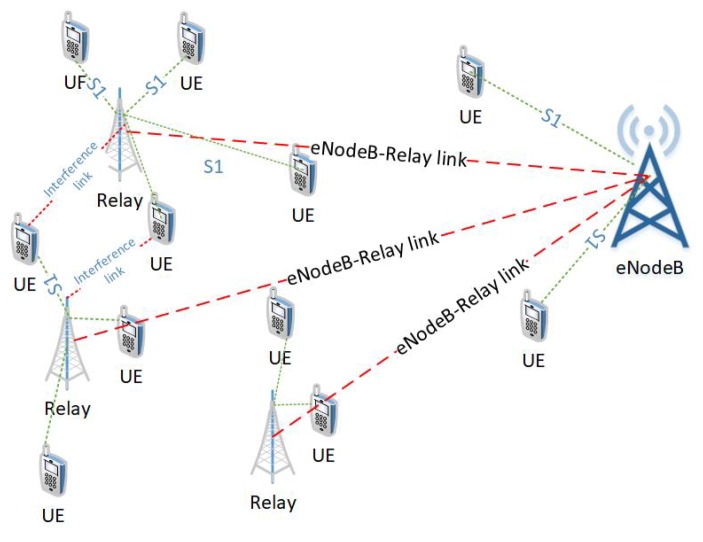
LTE-A Network with Multiple L3 Relays.

In practical scenarios, multiple relays are deployed to facilitate the cell-edge users as shown in the Figure 7. The multiple relay deployment causes inter-relay interference. This interference can be minimized by the careful deployment of relays, transmit power control, and the scheduling of time/frequency resources. Though, L3 relays incur more processing delay as compared to the L1 and L2 relays but they provide robust transmission in the presence of interference [26]. Assume there are *Q* relays in a cell, such that relay q∈Q={1,…,Q}. The signal-to-interference-and-noise ratio (SINR) at UE *k* in direct link is given as

(20) $$SINR_{k,n}^{Direct\_link}=\frac{p_{k,n}^{BS}g_{k,n}^{Direct\_link}}{\sum_{q\in Q}p_{k^{\prime },n}^{q}g_{k,n}^{q}+N_{0}\frac{B}{N}}$$

where $p_{k^{\prime },n}^{q}$ is the transmit power of relay *q* assigned to its associated user $k^{\prime }$ and $g_{k,n}^{q}$ is the channel gain between relay *q* and the UE *k*. Similarly, the SINR at UE *k* in relay *q* link is given as

(21) $$SINR_{k,n}^{q}=\frac{p_{k,n}^{q}g_{k,n}^{q}}{\sum_{q^{\prime }\in Q-\left\{q\right\}}p_{k^{\prime },n}^{q^{\prime }}g_{k,n}^{q^{\prime }}+p_{k,n}^{BS}g_{k,n}^{Direct\_link}+N_{0}\frac{B}{N}}$$

**Figure 8:**
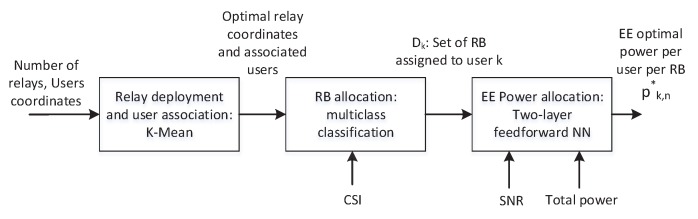
Block diagram of machine learning based resource and power allocation.

As seen from the above equation, the interference and fairness causes a significant increase in the computational cost when deploying multiple relays. Therefore, we present a machine learning based approach that utilizes relay deployment and users’ association data to develop RB allocation and Power allocation strategy that maximizes the sum EE. Once trained, the proposed approach can save cost of scheduling in every TTI. This is shown in Figure 8, the machine learning model takes the inputs: number of relays, relays’ coordinates, CSI, SNR, and total transmit power and produces the outputs: optimal relays’ coordinates with associated users, set of RBs assigned to each user *k*, and the optimal power allocation ($p_{k,n}^{*}$) to each user *k* in the RB *n*. Based on single relay performance, the RB allocation block is trained using SAMM and power allocation block is trained using BOPA. Since the relay deployment can significantly alter the RB and power allocation, a clustering approach is presented that determines relay positioning and corresponding users’ association based on a pre-defined metric.

### 4.1. Relays Deployment and Users Association

In this section, we present an autonomous unsupervised machine learning scheme that provides users association with optimally deployed relay nodes in the cell-edge area. Machine learning algorithms can broadly be divided into two main categories, namely supervised learning and unsupervised learning algorithms. The former class of algorithms learn by training on the input labeled examples, called training dataset, $\left\{\left(x^{\left(1\right)},y^{\left(1\right)}\right),\left(x^{\left(2\right)},y^{\left(2\right)}\right),\left(x^{\left(3\right)},y^{\left(3\right)}\right),\ldots ,\left(x^{\left(m\right)},y^{\left(m\right)}\right)\right\}$, where the $i^{th}$ example $\left(x^{\left(i\right)},y^{\left(i\right)}\right)$ consists of the $i^{th}$ instance of feature vector $x^{\left(i\right)}$ and the corresponding label $y^{\left(i\right)}$. Given a labeled training dataset, these algorithms try to find the decision boundary that separates the positive and negative labeled examples by fitting a hypothesis to the input dataset. Unsupervised machine learning algorithms, on the other hand, are given an unlabeled input dataset. These algorithms are used for extracting information or features from the dataset. These features might be related, but not confined, to the underlying structures or patterns in the input data, relationships in data items, grouping/clustering of data items, etc. Discovered features are meant to provide a deeper insight into the input dataset that can subsequently be exploited for achieving specific goals. Clustering algorithms make an important part of unsupervised learning where the input examples are grouped into two or more separate clusters based on some features. The K-Means (KM) algorithm, is probably the most popular clustering algorithm. It is an iterative algorithm that starts with a set of initial centroids given to it as input. During each iteration, it performs the following two steps.

1. **Assign Cluster:** For every user, the algorithm computes the distance between the user and every centroid. The user is then associated to the cluster with the closest centroid. During this step, a user might change its association from one cluster to another one.
2. **Recompute centroids:** Once all users have been associated to their respective cluster, the new position of centroid for every cluster is then calculated.

Let us define the following notations to be used later in this section.

$$\begin{array}{cc} K & =\mathrm{Total}\;\mathrm{number}\;\mathrm{of}\;\mathrm{clusters}\;\mathrm{being}\;\mathrm{formed}.\\ x^{\left(i\right)} & =\mathrm{Location}\;\mathrm{coordinates}\;\mathrm{of}\;\mathrm{user}\;u^{\left(i\right)}.\;\mathrm{In}\;\mathrm{our}\;\mathrm{case},\;x^{\left(i\right)}\in I\;R^{2}\\ c^{\left(i\right)} & =\mathrm{Cluster}\;\mathrm{to}\;\mathrm{which}\;\mathrm{the}\;\mathrm{user}\;u^{\left(i\right)}\;\mathrm{is}\;\mathrm{currently}\;\mathrm{associated}.\\ \mu_{k} & =\mathrm{Centroid}\;\mathrm{of}\;k^{th}\;\mathrm{cluster},\;\mu_{k}\in I\;R^{2}\;\\ \mu_{c^{\left(i\right)}} & =\mathrm{Centroid}\;\mathrm{of}\;\mathrm{the}\;\mathrm{cluster}\;\mathrm{to}\;\mathrm{which}\;\mathrm{the}\;\mathrm{user}\;u^{\left(i\right)}\;\mathrm{is}\;\mathrm{currently}\;\mathrm{associated}. \end{array}$$

Now the cost function *J* can be defined as

(22) $$J\left(c^{\left(1\right)},c^{\left(2\right)},\ldots ,c^{\left(m\right)},\mu_{1},\mu_{2},\ldots ,\mu_{K}\right)=\frac{1}{m}\sum_{i=1}^{m}\left|\right|x^{\left(i\right)}-u_{\left(c^{i}\right)}{\left|\right|}^{2}$$

with the following optimization objective function.

$$\begin{array}{c} \min_{c^{\left(1\right)},,\ldots ,c^{\left(m\right)},\mu_{1},\ldots ,\mu_{K}}J\left(c^{\left(1\right)},c^{\left(2\right)},\ldots ,c^{\left(m\right)},\mu_{1},\mu_{2},\ldots ,\mu_{K}\right) \end{array}$$

It may be pointed out that Equation (22) allows us to compare multiple clustering layouts based on their cost and select the one with the lowest cost.

In this section, we use the KM algorithm for optimal clustering of *m* users competing for resources in a particular cell. The clustering is performed based on their geographic location, thus our input dataset $\left\{u^{\left(1\right)},u^{\left(2\right)},u^{\left(3\right)},\ldots ,u^{\left(m\right)}\right\}$ has *m* vectors $u^{\left(i\right)},\;1\leq i\leq m$, consisting of location coordinates, of *i*th user. For the sake of simplicity, we assume these users are deployed in a two dimensional area, i.e., a plane and so $u^{\left(i\right)}=\left(x_{1}^{\left(i\right)},x_{2}^{\left(i\right)}\right)$, i.e., an ordered pair of location coordinates. Our clustering algorithm is summarized in Algorithm 2.

**Figure 9:**
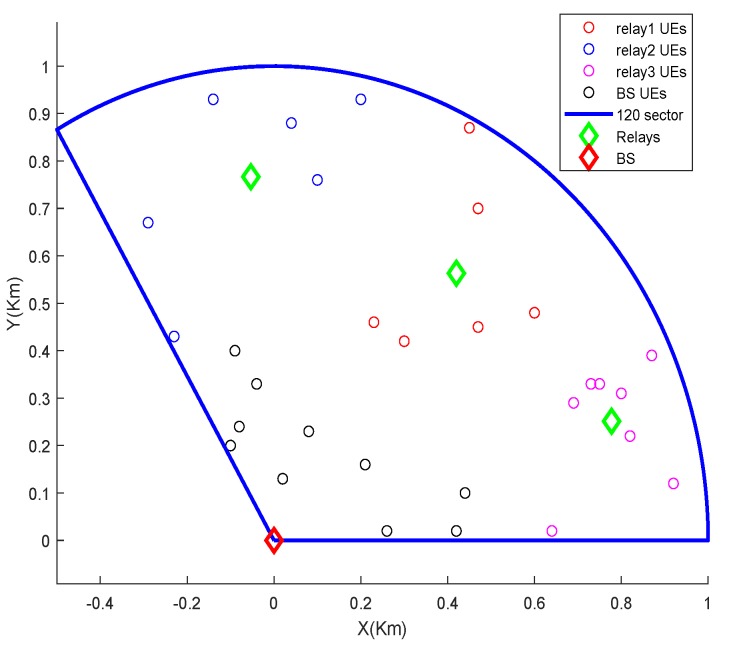
A snapshot of the relay deployment and users’ association algorithm output in a 120 degree sector.

The proposed algorithm takes the location coordinates of *m* users as input. It also takes two numbers $min_{k}$ and $max_{k}$ as additional inputs. The algorithm outputs the best number of clusters, *k*, such that $min_{k}\leq k\leq max_{k}$, and corresponding members of each cluster. It starts with $k=min_{k}$ and randomly selects *k* user locations as the initial centroids (line 6). It assigns the closest centroid to each user (line 8) and then computes new centroids by calculating the center/average location of all nodes in each cluster (line 11). So, in effect, the location of centroids keeps moving in successive iterations. It repeats the above two steps until the change in centroids’ positions is zero or negligible. We repeat the test $max_{t}$ times with a new set of randomly chosen initial centroids every time. During every test, the discovered centroids, corresponding centroid assignment to users, and the cost are saved (lines 14–16) for later comparison. After running the loop for $max_{t}$ times, we select and store the best *k* centroids resulting from the test with the lowest cost while discarding the remaining (lines 19–21). The same is repeated for the next value of *k*, i.e., k=k+1, until $k>max_{k}$. At the end we have $cnt=max_{k}-max_{k}$ vectors $\mu_{k}$, one for each value of *k*, the corresponding assignment vector $a_{k}$ and cost $c_{k}$. Finally, we choose the vector μ having the lowest cost and corresponding assignment vector a among cnt stored cases. That is the best number of clusters and corresponding centroids that the algorithm found. A snapshot of the relay deployment and users’s association algorithm output is shown in Figure 9.

**Algorithm 2** Users association clustering algorithm
1:
cnt=0
2:
**for** $k=min_{k}:max_{k}$ **do**
3: cnt=cnt+14: **for** $t=1:max_{t}$ **do**5:  **repeat**6:   Randomly choose initial *k* centroids $\mu_{1},\mu_{2},\mu_{3},\ldots ,\mu_{k}$
7:   **for** i=1:m **do**8:    $a^{\left(i\right)}=j,\;1\leq j\leq k$, such that $\mu_{j}$ is the centroid closest to $u^{\left(i\right)}$9:   **end for**10:   **for** l=1:k **do**11:    $\mu_{l}=$ mean of all users/points $u^{\left(i\right)}$ assigned to lth centroid12:   **end for**13:  **until** converges14:  $\mu^{\left(t\right)}=\left(\mu_{1},\mu_{2},\mu_{3},\ldots ,\mu_{k}\right)$15:  $a^{\left(t\right)}=\left(a^{\left(1\right)},a^{\left(2\right)},a^{\left(3\right)},\ldots ,a^{\left(m\right)}\right)$16:  $c^{\left(t\right)}=cost\left(\mu_{1},\mu_{2},\mu_{3},\ldots ,\mu_{k}\right)$17: **end for**18: $idx=\arg_{min}\left\{c\left(t\right),1\leq t\leq max_{t}\right\}$19: $\mu_{k}{}^{\left(k\right)}=\mu^{\left(idx\right)},1\leq idx\leq max_{t}$20: $a_{k}{}^{\left(k\right)}=a^{(idx),}1\leq idx\leq max_{t}$21: $c_{k}^{\left(k\right)}=c^{\left(idx\right)},1\leq idx\leq max_{t}$22:
**end for**
23:
$index=\arg_{min}\left\{c_{k}^{\left(k\right)},1\leq k\leq cnt\right\}$
24:
$\mu =\mu_{k}{}^{\left(index\right)},1\leq index\leq cnt$
25:
$a=a_{k}{}^{\left(index\right)},1\leq index\leq cnt$
26:
n=index



### 4.2. Resource Allocation by Multiclass Classification

**Figure 10:**
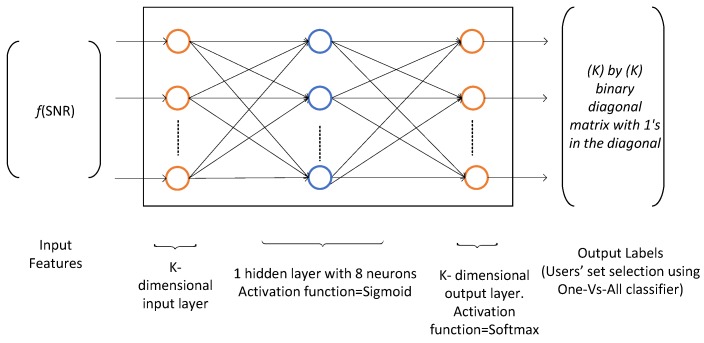
Neural network architecture for RB allocation, K=10.

The resource block allocation problem has multiple discrete outputs, i.e., the users, therefore, we use the multiclass classification to classify one out of *K* users. The multiclass classification is an extension of One-Vs-All classification. The input of the training network comprises of channel state information in terms of the SNR and the output consists of a particular user that maximizes the utility function (throughput for MT and PF metric for the proportional fairness). The training data is obtained from the implementation of SAMM algorithm of [4] as 25,000 K-dimensional samples of received SNR and the corresponding selected users. The dataset is partitioned into three parts, the training dataset, the validation dataset, and the test dataset. These are divided in 70%, 15%, and 15% ratio, respectively. The Matlab Neural Network Pattern Recognition Apps is used to train and deploy the neural network. It uses Scaled Conjugate Gradient algorithm [27] for training. Our application requires K=10 neurons in input layer and 10 neurons in output layer. A hit and trial choice of eight neurons in hidden layer gave the best result. The neural network architecture is shown in Figure 10.

The neural network loss function is a generalization of the logistic regression’s loss function. In logistic regression classification problem, we try to find the weighted parameter θ, such that the mean square error between the predicted output and the actual output is minimized. This is called loss function (LF) or the cost function and is given by

(23) $$LF\left(\theta \right)=\frac{1}{m}\sum_{i=1}^{m}{\left(h_{\theta }\left(x^{\left(i\right)}\right)-y^{\left(i\right)}\right)}^{2}$$

where the prediction or hypothesis function $h_{\theta }\left(x\right)$ is a sigmoid function, i.e., $h_{\theta }\left(x\right)=\frac{1}{1+e^{-\theta^{T}x}}$. In the above equation, $\left(x^{\left(i\right)},y^{\left(i\right)}\right)$ is a training dataset with 1,…,m input-output pairs. However, loss function with sigmoid function leads to a non-convex function, therefore, a cross entropy based loss function is used to make it convex function as,

(24) $$LF\left(\theta \right)=-\frac{1}{m}\sum_{i=1}^{m}\left[y^{\left(i\right)}\log\left(h_{\theta }\left(x^{\left(i\right)}\right)\right)+\left(1-y^{\left(i\right)}\right)\log\left(1-h_{\theta }\left(x^{\left(i\right)}\right)\right)\right]+\frac{\lambda_{R}}{2m}\sum_{j=1}^{n}\theta_{j}^{2}$$

where the second summation is for the regularization of weight or bias units $\theta_{j}$ and $\lambda_{R}$ is a regularization parameter.

In case of neural networks with multiclass classification, the prediction variable becomes K-dimension, $h_{\Theta }\left(x\right)\in R^{K}$, therefore, the loss function is given as

(25) $$LF\left(\Theta \right)=-\frac{1}{m}\sum_{i=1}^{m}\sum_{k=1}^{K}\left[y_{k}^{\left(i\right)}\log{\left(h_{\Theta }\left(x^{\left(i\right)}\right)\right)}_{k}+\left(1-y^{\left(i\right)}\right)\log\left(1-{\left(h_{\Theta }\left(x^{\left(i\right)}\right)\right)}_{k}\right)\right]+\frac{\lambda_{R}}{2m}\sum_{l=1}^{L-1}\sum_{i=1}^{s_{l}}\sum_{j=1}^{s_{l+1}}{\left(\Theta_{j,i}^{\left(l\right)}\right)}^{2}$$

where *L* is the number of layers in neural network, $s_{l}$ is the number of neurons in layer *l*, and $\lambda_{R}=5\times 10^{-4}$ is a regularization parameter to control the tradeoff between fitting the training dataset and keeping the parameter Θ small. The neural network is trained using the stochastic gradient descent algorithm. The gradient or partial derivative is calculated by the backpropagation algorithm and weights (θ) are updated. The amount at which the weights are updated is called learning rate. It our case, we set learning rate to 0.01. Batch size is a matrix of input (or output) vectors applied to the network simultaneously to produce the update on network weights and biases. In our work, batch size of 128 (MATLAB default), 10×1 input vectors is used.

We use MATLAB 2019a App, Neur al Network Pattern Recognition (nprtool) which is a two-layer (one for hidden layer activation functions and other for output layer activation functions) feedforward network.

**Figure 11:**
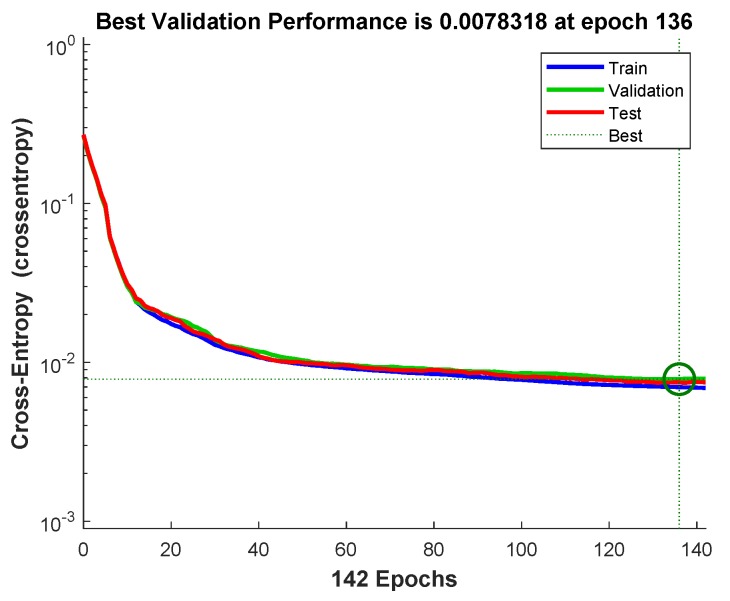
The mean-squared-error for the training and testing of the RB allocation module.

**Figure 12:**
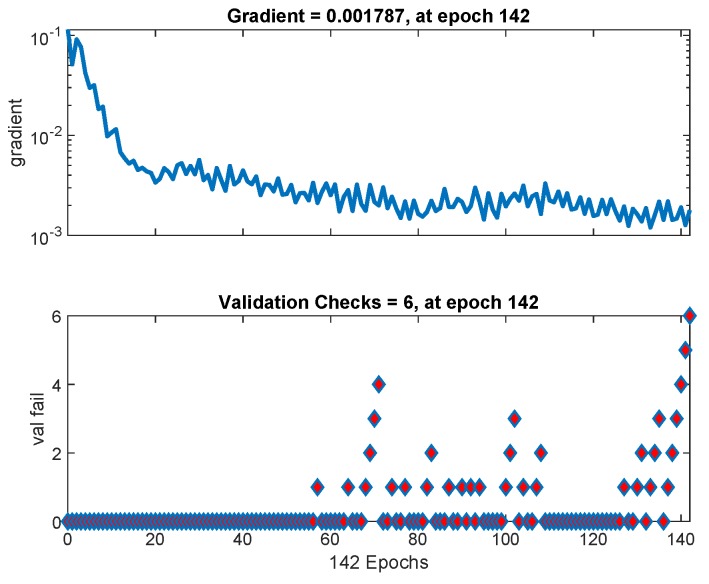
The neural network training states with gradient and validation fail statistics as a function of number of epochs.

Lower the cross entropy higher the classification accuracy, zero cross entropy means no error. Figure 11 shows that cross entropy reaches 0.0078318 at iteration 136. Figure 12 shows variation in gradient coefficient with respect to number of epochs. The final value of gradient coefficient at epoch number 142 is 0.001787 which is approximately near to zero. Minimum the value of gradient coefficient better will be training and testing of networks. From the figure, it can be seen that the gradient value is decreasing with the increase in number of epochs. Large number of validation fails indicate the overtraining. In Figure 12 validation fails are the iterations when validation mean square error (MSE) increased its value. A lot of fails means overtraining. MATLAB automatically stops training after 6 fails in a row.

**Figure 13:**
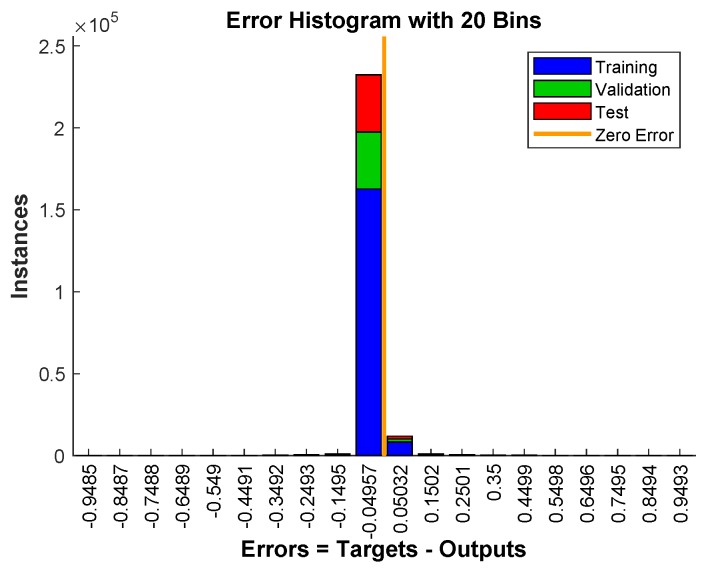
The error histogram of the trained neural network for the training, validation and testing phases.

**Figure 14:**
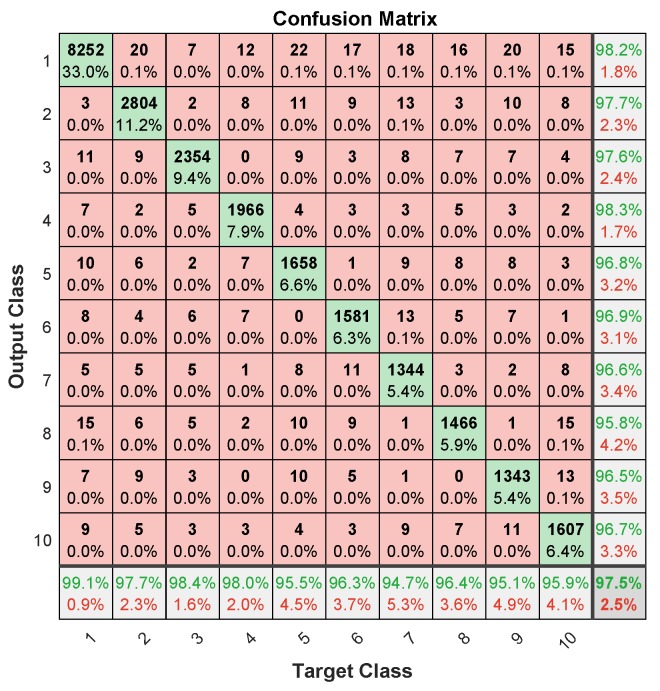
The confusion matrix for test dataset.

**Figure 15:**
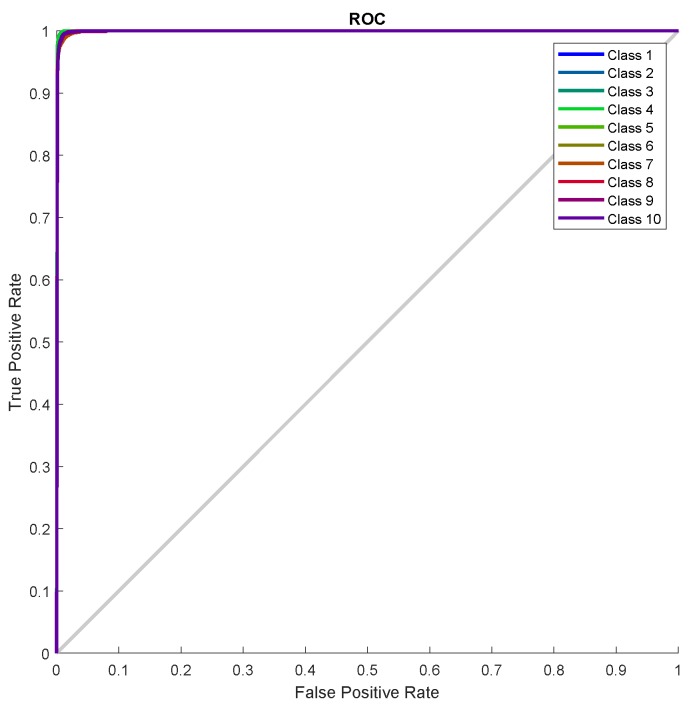
Receiver Operating Characteristic (ROC curve).

Figure 13 shows the error histogram of the trained neural network for the training, validation and testing parts. In this figure we can see that the data fitting errors are minimum and they are distributed within a closed range around zero. The confusion matrix Figure 14 visualizes the performance of supervised learning. The rows correspond to the predicted user (Output Class) and the columns correspond to the true user (Target Class). The diagonal cells correspond to observations that are correctly assigned the user-RB pairs. The off-diagonal cells correspond to incorrectly assigned user-RB pairs. The trained neural network provides 97.5% classification accuracy. The Figure 15 represent the receiver operating characteristics (ROC) curves. The ROC curve plot shows the true positive rate versus the false positive rate as the threshold is varied. A perfect test would show points in the upper-left corner, with 100% sensitivity and 100% specificity [28]. In the RB allocation module, it worked very well.

### 4.3. Power Allocation through Two-Layer Feedforward Neural Network

**Figure 16:**
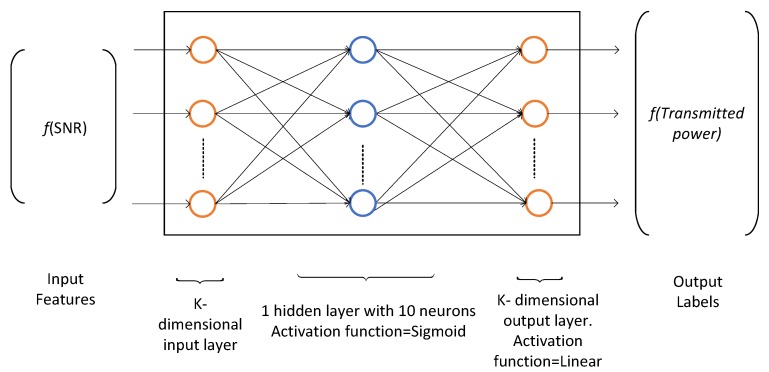
Neural network architecture for power allocation, K=10.

In the power allocation problem, we have to map the numeric input dataset (SNR) to the numeric output dataset (allocated power) per user per RB. Therefore, we use neural network curve fitting technique. The training dataset is generated by the Algorithm 2 as input received SNR and output allocated transmit power. Given the resource blocks allocation set $D_{k}\;\forall k\in K$, the power allocation problem has been solved using two-layer feedforward neural network. The hidden layer neurons use sigmoid function as activation function and output neurons implement linear function as shown in Figure 16. We use Bayesian Regularization method to train the neural network. This method typically requires more training time but gives good results for difficult and noisy dataset. The Bayesian Regularization method uses Levenberg-Marquardt optimization to update the weight and bias values. It minimizes a combination of squared errors and weights, and then determines the correct combination for better generalization. In this method, the training does not stops after six consecutive validation (improve) fails and by default max_fail = inf. The training continues until an optimal combination of errors and weights is reached. More detail on the use of Bayesian regularization, along with Levenberg-Marquardt training, can be found in [29].

We use MATLAB 2019a App, Neural Net Fitting (nftool) which is a two-layer (one for hidden layer activation functions and other for output layer activation functions) feedforward network.

**Figure 17:**
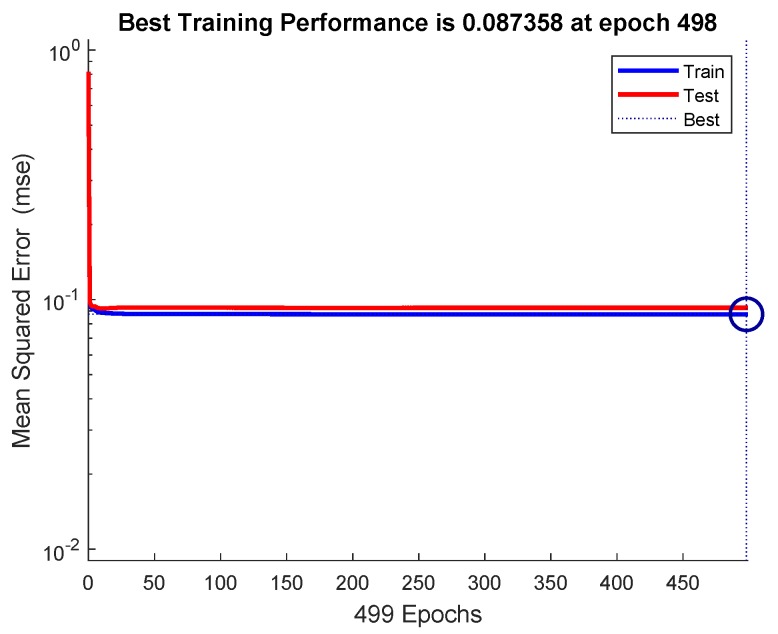
The mean-squared-error for the training and testing of the power allocation module.

**Figure 18:**
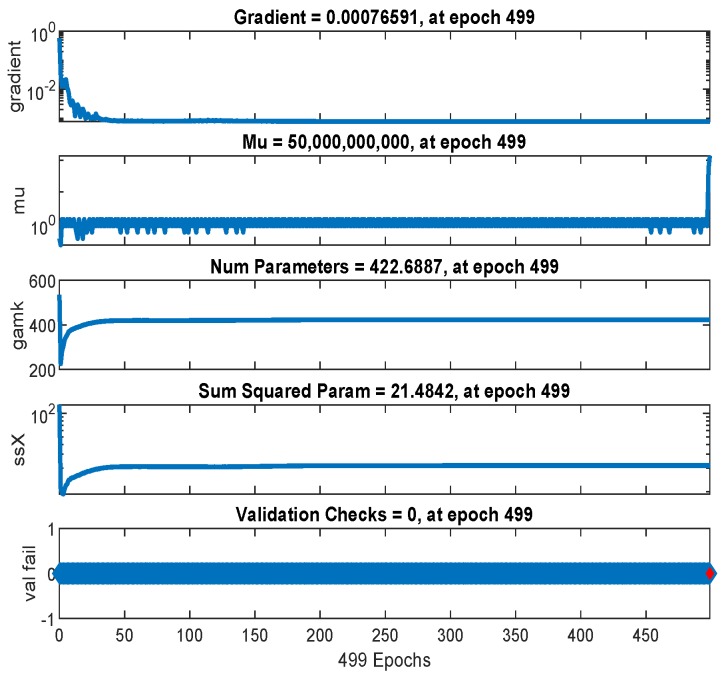
The neural network training states.

**Figure 19:**
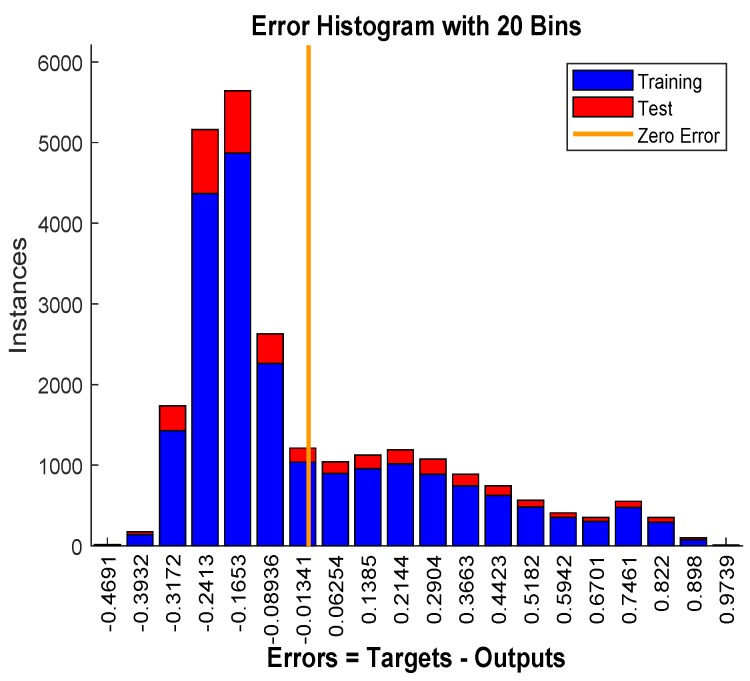
The error histogram.

The mean-squared-error graph for the training and testing is shown in Figure 17. It shows that the MSE reaches to 0.087358 in 498 epochs. Our input/output samples to training network were channel gain/allocated power. Since, the total transmit power is a sum of linear functions of the channel gain, therefore, the neural network is got trained in a single epoch. An epoch is a full pass through the entire dataset and the calculation of new weights and biases. Figure 18 shows that the gradient coefficient reaches to 0.00076591 in 499 epochs. The lower value of gradient ensures the training and testing of the network. Other parameters such as Mu, Num Parameters, and Sum Squared Param are the stop criteria defined in Bayesian regularization backpropagation function ’trainbr’ [30]. Error histogram in Figure 19 visualizes the errors between target values and the predicted values after training a feedforward neural network. In this figure we can see that the data fitting errors are minimum and they are distributed within a closed range around zero. Around 88.1% errors fall between −0.3 and 0.33.

### 4.4. Performance Evaluation with Machine Learning Techniques

**Figure 20:**
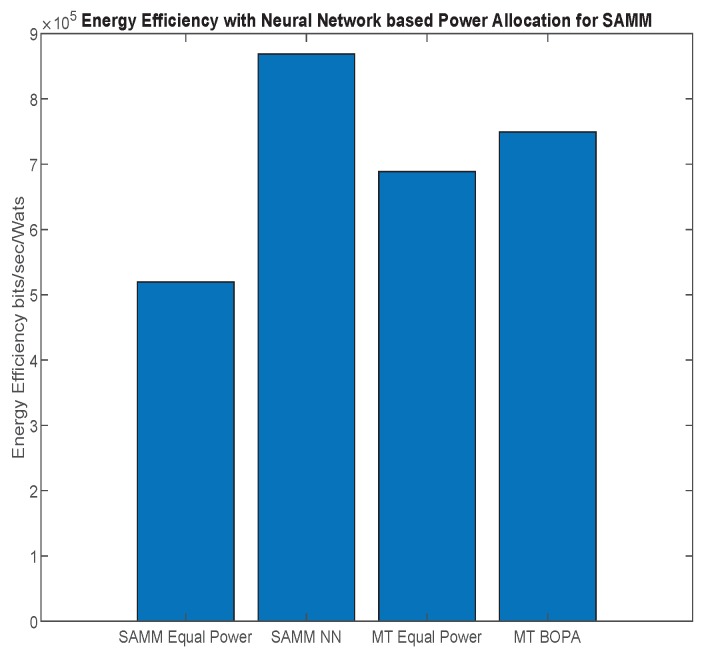
The system energy efficiency with neural network for SAMM which is trained on waterfilling based power allocation among users and BOPA based power allocation among subchannels in a single relay scenario.

**Figure 21:**
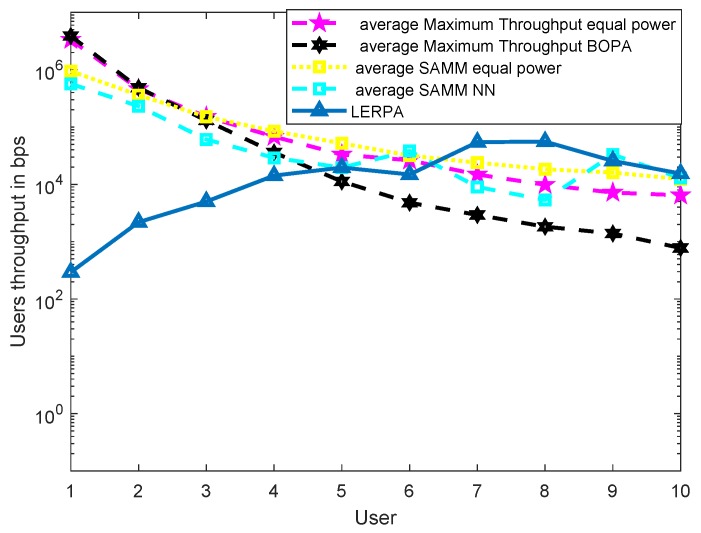
The users’ throughput with neural network for SAMM along with LERPA of [11] in multiple relays scenario.

**Figure 22:**
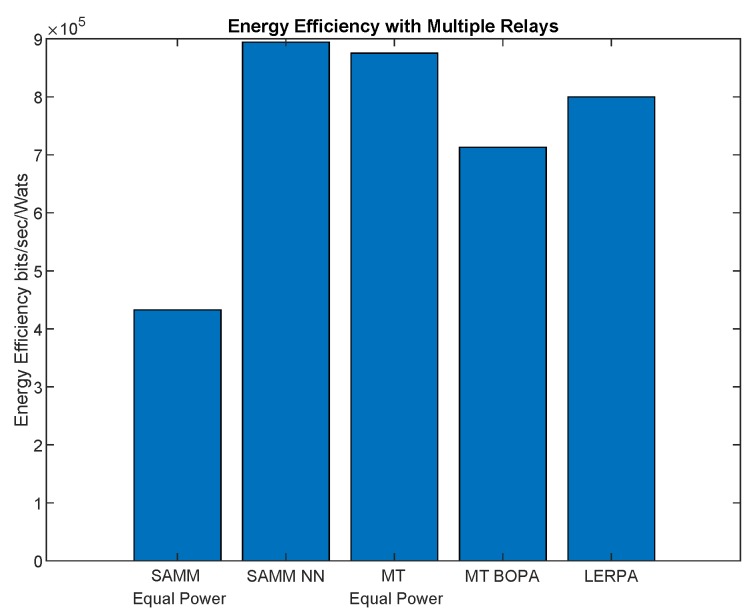
The system energy efficiency with neural network for SAMM which is trained on waterfilling based power allocation among users and BOPA based power allocation among subchannels along with LERPA of [11] in a multiple relays scenario.

First, we apply the neural network for the RB and power allocation modules with a single relay. For the SAMM scheme in Figure 20 shows 30.25% increase in the EE. This is because of the limitations of the BOPA method which sometimes returns no result, whereas, the neural network is trained on diverse dataset and always gives the output result. We also compare our proposed schemes with LERPA of [11]. LERPA uses max–min criteria for RB allocation and fractional programming based transmission power control. In case of LTE network with multiple relays as shown in Figure 7 or Figure 9, the users associated with relay *q* experience interference due to the neighboring relays $q_{neigh}$. This interference decreases the users’ throughput as shown in Figure 21. However, the EE maximization based NN power allocation continues to dominate in the multiple relay scenario. Since the transmission is orthogonal between BS and RNs, only the relay’s associated users are affected by the other relays transmissions. The equal power MT throughput does not affect because almost all the users are associated with BS. This further reduces the required transmit power of the relay, hence a net increase in EE has been observed in Figure 22. Addition of multiple relays slightly affect the SAMM NN and SAMM equal power in positive and negative way, respectively. The PF component of the SAMM forces the association of low throughput users to increase the fairness. This association goes in positive way for the SAMM NN due to the EE based power allocation, but goes in negative way for the SAMM equal power because of no compensation of the interference power. The increased fairness of SAMM NN is evident from the Figure 21, where, even the farthest users 9 and 10 have higher throughput. It can be seen that in LERPA, closer users get lower throughput but fairly large throughput is given to the farther users. This is because it uses max-min criterion for the RB allocation, which assigns the RB to the users who have lowest received SNR.

Table 3 summarizes simulation results.

It can be seen that SAMM with BOPA and NN compete well in fairness with best EE. Tradeoff has to be done on system throughput. LERPA has better fairness performance but is less efficient in EE and system throughput, whereas, the hypothetical MT performs better in average system throughput. We say hypothetical because it only allocates the RB and power to the users with the highest SNR which can not be applicable on practical scenarios.

## 5. Complexity Analysis

The RB allocation scheme SAMM uses alternate MT and PF metrics to assign the *N* RBs to *K* users. MT assigns $\frac{N}{2}$ RBs to *K* users and PF assigns $\frac{N}{2}$ RBs to K−1 users in alternate TTI. Therefore, the computational complexity of SAMM is $O\left(N(K-\frac{1}{2})\right)$. The BOPA Algorithm 1, first requires $\lambda_{min}$ and $\lambda_{max}$ in line 4 using water-filling algorithm for which the worse-case complexity is O(2NK). After that, BOPA uses binary search method to estimate the roots of Equation (17). In the worse case, with $N_{p}$ points in the search space, binary search requires $\log_{2}\left(N_{p}\right)$ iterations to find the roots of polynomial. In our case, $N_{p}=\frac{\lambda_{max}-\lambda_{min}}{\epsilon }$, where ϵ is the error tolerance. Therefore, the overall complexity of the Algorithm 1 is $O(2NK^{2}\log_{2}\left(N_{p}\right))$. In case of the optimal exhaustive search ($K^{N}$) RB allocation combining with the BOPA; the complexity is $O(2NK^{N+2}\log_{2}\left(N_{p}\right))$, whereas, the complexity of SAMM-BOPA is $O({\left(NK\right)}^{2}\left(2K+1\right)\log_{2}\left(N_{p}\right))$.

The running-time complexity of the K-mean algorithm is O(kmdi) [31], where *k* is the number of clusters, *m* is the number of objects to be clustered, *d* is the dimension of objects, and *i* is the number of iterations. In our application of K-mean Algorithm 2, we use $min_{k}<k<max_{k}$ and two-dimensional geographical location of the users. Therefore, the worse-case computational complexity is given as $O(max_{k}Ki)$.

## 6. Conclusions

In this paper, we have investigated the impact of using single and multiple L3 relays in terms of EE and throughput. For a single relay scenario, equal power and BOPA are used in conjunction with the SAMM and MT RB allocation algorithms. Simulation results show that SAMM BOPA has 26% power saving when compared with MT BOPA. Whereas, when comparing SAMM with equal power allocation to all RBs, our proposed scheme gives 77% increase in EE. For a multiple relay scenario, a clustering scheme is proposed that addresses relay placement and users’ association. This information acts as an input to a machine learning process (SAMM NN) that cognizes both the SAMM and BOPA approaches using One-Vs-All classification and feedforward neural networks, respectively. The SAMM NN approach when compared with the SAMM Equal Power, gives a 2.07 times increase in EE at the cost of 0.72 times decrease in throughput. A SAMM BOPA approach adopted in the case of single relay still provided the best tradeoff in terms of energy efficiency EE, throughput and fairness in the case of multiple relays.

## Figures and Tables

**Table 1:** List of notations.

Notation	Definition
$\mu_{k,n}$	the RB assignment indicator
$R_{k}$	capacity of user *k* is given by the Shannon Capacity
$SNR_{k,n}$	the signal-to-noise ratio for user *k* on RB *n*
*B*	System bandwidth
*N*	Number of RB
$W=\frac{B}{N}$	the RB bandwidth
$P_{total}^{RN}$, $P_{total}^{BS}$	the total power at which RN and BS transmit
$g_{k,n}^{Relay\_link}$, $g_{k,n}^{Direct\_link}$	channel gains for user *k* on RB *n* for RN and BS
$h_{k,n}^{Relay\_link}$, $h_{k,n}^{Direct\_link}$	random channel coefficients for user *k* on RB n for RN and BS
$SNR_{k,n}^{Direct\_link}$	signal-to-noise ratio for user *k* via Direct Link
$SNR_{k,n}^{Relay\_link}$	signal-to-noise ratio for user *k* via Relay Link
$SINR_{k,n}^{Direct\_link}$	signal-to-Interference-and-noise ratio for user *k* via Direct Link
$\alpha_{k}$	proportional rate constraint for user *k*
$\lambda_{k}$	rate parameter for user *k*
$D_{k}$	allocated RB set for user *k*
$R_{k}$	rate matrix for user *k*
${\hat{p}}_{k,n}$	optimal power allocation
$P_{T}$	total transmit power
θ	Lagrangian multiplier

**Table 2:** Simulation Parameters.

Parameter	Value
Cell Radius	1 Km
Noise Density (σ)	−171 dBm/Hz
No of users (K)	10
Bandwidth (B)	5 MHz
Number of resource blocks (RB) (N)	25
No of subcarriers per RB	12
Subcarrier bandwidth	15 KHz
BS Transmitter Power	46 dBm
Relay Power	34 dBm
TTI duration	0.5 ms
Relay distance from BS	0.55 Km
Bit Error Rate (BER)	$10^{-3}$
OFDM symbols per TTI	7
Relay Type (In-band / Out-band Type 1 / Type 2)	In-band with Type 1b (full duplex)

**Table 3:** Simulation Results.

KPI	SAMM Equal Power	SAMM BOPA	SAMM NN	MT Equal Power	MT BOPA	LERPA [11]
Energy Efficiency (Mbps/Watts)	0.5128	0.5481	1.0630	0.6660	0.5202	0.8862
System average throughput (Mbps)	2.1471	1.1845	1.1037	4.0778	4.5775	0.4137
Throughput fairness index	0.3155	0.2337	0.2234	0.1453	0.1366	0.5797

## Abbreviations

The following abbreviations are used in this manuscript:

BER	Bit Error Rate
BOPA	Bisection based Optimal Power Allocation
BS	Base Station
CA	Carrier Aggregation
CoMP	Coordinated Multipoint
CSI	Channel State Information
EE	Energy Efficiency
JFI	Jains Fairness Index
L3	Layer 3
LTE-A	Long Term Evolution Advanced
MIMO	Multiple-input multiple-output
MQAM	M-ary Quadrature Amplitude Modulation
MT	Maximum Throughput
OFDM	Orthogonal Frequency Division Multiplexing
PF	Proportional Fairness
QoS	Quality of service
RB	Resource Block
RN	Relay Node
RR	Round Robin
SAMM	hybrid proportional fairness scheme
SINR	signal-to-interference-and-noise ratio
SNR	signal-to-noise ratio
TTI	Transmission Time Interval
UE	User Equipment
